# Identification of 22 Novel Motifs of the Cell Entry Fusion Glycoprotein B of Oncolytic Herpes Simplex Viruses: Sequence Analysis and Literature Review

**DOI:** 10.3389/fonc.2020.01386

**Published:** 2020-08-19

**Authors:** Fang Shi, Victoria W. Xin, Xiao-Qin Liu, Ying-Ying Wang, Ying Zhang, Jun-Ting Cheng, Wen-Qi Cai, Ying Xiang, Xiao-Chun Peng, Xianwang Wang, Hong-Wu Xin

**Affiliations:** ^1^Laboratory of Oncology, Center for Molecular Medicine, School of Basic Medicine, Health Science Center, Yangtze University, Jingzhou, China; ^2^Department of Biochemistry and Molecular Biology, School of Basic Medicine, Health Science Center, Yangtze University, Jingzhou, China; ^3^Department of Gastroenterology, Huanggang Central Hospital, Huanggang, China; ^4^Department of Biology, School of Humanities and Sciences, Stanford University, Stanford, CA, United States; ^5^Department of Pathophysiology, School of Basic Medicine, Health Science Center, Yangtze University, Jingzhou, China; ^6^Department of Laboratory Medicine, School of Basic Medicine, Health Science Center, Yangtze University, Jingzhou, China; ^7^Lianjiang People's Hospital, Guangdong, China

**Keywords:** oncolytic herpes simplex virus (oHSV), glycoprotein B (gB), motif, domain, cancer

## Abstract

**Objective:** Herpes simplex viruses (HSVs) are widely spread throughout the world, causing infections from oral, and genital mucous membrane ulcerations to severe viral encephalitis. Glycoprotein B (gB) was the first HSV envelope glycoprotein identified to induce cell fusion. This glycoprotein initiates viral entry and thereby determines the infectivity of HSV, as well as oncolytic HSV (oHSV). Clarifying its molecular characterization and enlarging its motif reservoir will help to engineer oHSV and in cancer treatment applications. Only in recent years has the importance of gB been acknowledged in HSV infection and oHSV engineering. Although gB-modified oHSVs have been developed, the detailed molecular biology of gB needs to be illustrated more clearly in order to construct more effective oHSVs.

**Method:** Here, we performed a systematic comparative sequence analysis of gBs from the 9 HSV-1 and 2 HSV-2 strains, including HSV-1-LXMW, which was isolated by our lab. Online software was implemented to predict gB secondary structure and motifs. Based on extensive literature reviews, a functional analysis of the predicted motifs was performed.

**Results:** Here, we reported the DNA and predicted amino acid sequences of our recently isolated HSV-1-LXMW and found that the strain was evolutionarily close to HSV-1 strains F, H129, and SC16 based on gB analysis. The 22 novel motifs of HSV gB were identified for the first time. An amino acid sequence alignment of the 11 HSV strains showed that the gB motifs are conserved among HSV strains, suggesting that they are functional *in vivo*. Additionally, we found that certain amino acids within the 13 motifs out of the 22 were reported to be functional *in vivo*. Furthermore, the gB mutants and gB-engineered oHSVs were also summarized.

**Conclusion:** Our identification of the 22 novel motifs shed light on HSV gB biology and provide new options for gB engineering to improve the efficiency and safety of oHSVs.

## Introduction

Cancer is a major threat to human health. Cancer stem cells play an important role in cancer initiation, progression, and drug resistance (1–5). Oncolytic viruses (OVs) are spontaneously occurring or genetically modified viruses that preferentially infect and kill cancer cells, including cancer stem cells (6–8). An oncolytic adenovirus with an E1B deletion, H101, or Oncorine®, was approved to treat cancer in China in 2005, which is the first clinical OV in the world (9). Recently, OVs, including the measles virus (MV), Newcastle disease virus (NDV), herpes simplex virus (HSV), reovirus, adenoviruses, and vesicular stomatitis virus (VSV), provide promising approaches in cancer treatment (10). Engineered HSV is considered a promising agent because of a series of unrivaled merits, including its opulent genome (150 kbp) and exhaustive cognition of viral genes (11). In 2015, the U.S. Food and Drug Administration (FDA) approved T-vec, an oncolytic HSV that was engineered with deletions of genes γ34.5 and US12 (encoding ICP47) and subsequent insertion of the gene encoding human granulocyte macrophage colony-stimulating factor (GM-CSF), to treat melanoma (12). Oncolytic HSVs (oHSVs) have also been made by engineering envelope glycoproteins (13).

HSV entry is a perplexing process, requiring the coordination of glycoprotein ligands gD, gB, gH, and gL alongside cellular receptors. These glycoproteins interact with cellular receptors and lead to virus–cell fusion (14). The cascade is initiated by HSV to heparan sulfate proteoglycans (HSPG) at the cell surface. While HSV-1 gC has a critical role in HSPG attachment during HSV-1 entry, HSV-2 gB is the key glycoprotein for HSV-2 attachment to HSPG (15, 16). gD attaches to one of its own receptors, herpesvirus entry mediator (HVEM), nectin-1, or 3-O-sulfated heparan sulfate (3-OS HS) (17–19), activating gH/gL and changing the conformation of gB (17). The attachment of gH/gL to its own receptors may also be involved in this process (17, 20). gB inserts itself into the cell membrane and is considered as a viral fusogen (21, 22). Virus–cell fusion can only take place when gB interacts with one of the cell's receptors. There are three HSV-1 gB receptors: the myelin-associated glycoprotein (MAG), the paired immunoglobulin-like type 2 receptor α (PILRA), and the myosin heavy chain 9 (MYH9). However, HSV-2 gB-specific receptors remain unknown.

gB is a conservative glycoprotein (23). HSV-1 gB is composed of four parts: a secretory signal, an extracellular region, a transmembrane region, and a cytoplasmic region (24). Furthermore, gB contains five domains: domain I contains residues Ile154 to Val363; domain II is composed of residues Tyr142 to Asn153 and Cys364 to Thr459; domain III is composed of residues Pro117 to Pro133, Ser 500 to Thr572, and Arg661 to Thr669; domain IV encompasses residues Ala111 to Cys116 and Cys573 to Ser 660; domain V ranges from residues Phe670 to Ala725 (23). Four functional regions of gB were identified alongside the domains: (i) the first region consists of domain I, residues Glu697 to Ala725, and contains two internal fusion loops (FLs); (ii) the second region, which is generated by Ile391 to Gly410, Pro454 to Lys475, and a part of domain II, may play a direct role in gH/gL binding; (iii) the third region includes residues within domains III and IV that bind to a cellular receptor; and (iv) the last region contains Met1 to Trp12 (25, 26).

However, the details of gB structure and function are still not fully understood (27). The efficiency of oHSV may be improved upon in the future. (22). Here, we sequenced the DNA encoded the gB of our recently isolated new strain HSV-1-LXMW; compared the gB amino acid sequence with 10 other gBs through phylogenetic analysis, amino acid sequence alignment, and motif predictions; and found 22 new motifs in HSV gB. We further correlated the newly identified motifs with engineered gB reported in HSVs. Our studies may enrich the knowledge on gB structure, and gB-engineered oHSV may contribute greatly to cancer treatment.

## Materials and Methods

### HSV Genomic DNA Sequencing Analysis

A new HSV strain, named HSV-1 strain LXMW, was obtained previously (28). Briefly, a 45-year-old male patient with acute oral herpes was admitted for HSV-1 isolation. The herpes lesion was punctured with a sterile syringe, the liquid was dipped onto sterile cotton swab, and the swab was placed in a virus collection tube [Youkang Keye Biotechnology (Beijing) Co., Ltd., Cat. No. MT0301] and shipped at 4°C to the laboratory. After successful isolation and identification, genomic HSV DNA sequencing was described in a previous publication (28). The high-quality genomic DNA (500 ng) was submitted to the Beijing Genomics Institute (BGI, http://www.genomics.cn). The *UL27* DNA sequence was presented in Supplementary Materials. Afterwards, the *UL27* DNA sequence was translated into gB amino acids using the DNAMAN software (http://www.shinegene.org.cn/q2.html). The translation result was also shown in Supplementary Materials. In addition, we applied the Burrows-Wheeler Aligner (BWA) software (https://github.com/lh3/bwa) to perform the alignment. The genomic sequences of 10 other HSV strains were obtained from NCBI Reference Database (Table 1).

**Table 1:** The information of HSV strains studied in this article.

**HSV Strain**	**Gene Bank ID**	**Tax-ID**	**Sub-Date**	***UL27* DNA sequence**	**University, Country**
HSV-1 strain LXMW				53088/55802	Yangtze University, Jingzhou, China
HSV-1 strain 17	JN555585.1	10299	2011-08-02	53059/55795	RC University, Glasgow, UK
HSV-1 strain H129	GU734772.1	744249	2010-2-9	53007/56030	Princeton University, USA
HSV-1 strain RH2	AB618031.1	946522	2011-2-28	51607/54321	Osaka University, Japan
HSV-1 isolate SC16	KX946970.1	10309	2016-10-30	53707/56443	Severo Ochoa, Spain
HSV-1 strain Patton Isolate GFP-Us11	MF959544.1	10308	2017-10-11	53071/56093	NYU, New York, USA
HSV-1 strain F	GU734771.1	10304	2010-2-9	52974/55996	Princeton University, USA
HSV-1 strain KOS	JQ673480.1	10306	2012-2-14	53022/55736	University of Kansas, USA
HSV-1 strain MP-R15	EF177455	10298	2007-10-09	1/2700	Goteborg University, Sweden
HSV-2 strain HG52	JN561323.2	10315	2011-08-05	53402/56152	University of Glasgow, UK
HSV-2 strain G	KU310668.1	10314	2015-12-16	1/2706	Einstein College, USA

### Phylogenetic Analysis of gB Amino Acids From HSV-1 and HSV-2

The software MEGA7 (http://www.megasoftware.net) was implemented for the phylogenetic analysis of gB amino acid sequences from 11 HSV strains. Based on the General Time Reversible model with “complete deletion,” the Maximum Likelihood method option was applied to infer evolutionary history. The evolutionary history of the taxa analyzed was represented in a bootstrap consensus tree. The percentage of trees, in which the associated taxa were clustered together, is displayed next to the branches. The cladogram with the highest log likelihood is displayed. The details of the method were described previously (28).

### Alignment of gB Amino Acid Sequences for 11 HSV Strains

gB amino acids were previously translated from the *UL27* DNA sequence using the DNAMAN software (http://www.shinegene.org.cn/q2.html). The 10 other amino acid sequences utilized for the analysis were translated from the above DNA sequences. To determine the conservation of gB amino acid sequences among different HSV strains, the online software EMBL-EBI (https://www.ebi.ac.uk) was implemented to perform the alignment of gB amino acid sequences. The stringent conditions were set as default by the online program. Aberrant amino acids were marked in red.

### Prediction of gB Secondary Structure and Motifs

Secondary structure and motifs are the basis for the formation of functional domains and usually play a crucial role in protein function. The online software UCL-CS Bioinformatics (http://bioinf.cs.ucl.ac.uk/introduction/) was deployed to predict the secondary structure of gB. Motifs were predicted through the online software MEME (http://meme-suite.org/). The stringent conditions were set as default by the online program. To show the results briefly and conveniently, one HSV-1 strain (HSV-1 strain KOS), one HSV-2 strain (HSV-2 strain G), and our new HSV-strain LXMW were selected.

## Results

### HSV-1-LXMW Is Evolutionarily Close to HSV-1 Strains F, H129, and SC16 Based on *UL27* DNA Sequence Analysis

**Figure 1:**
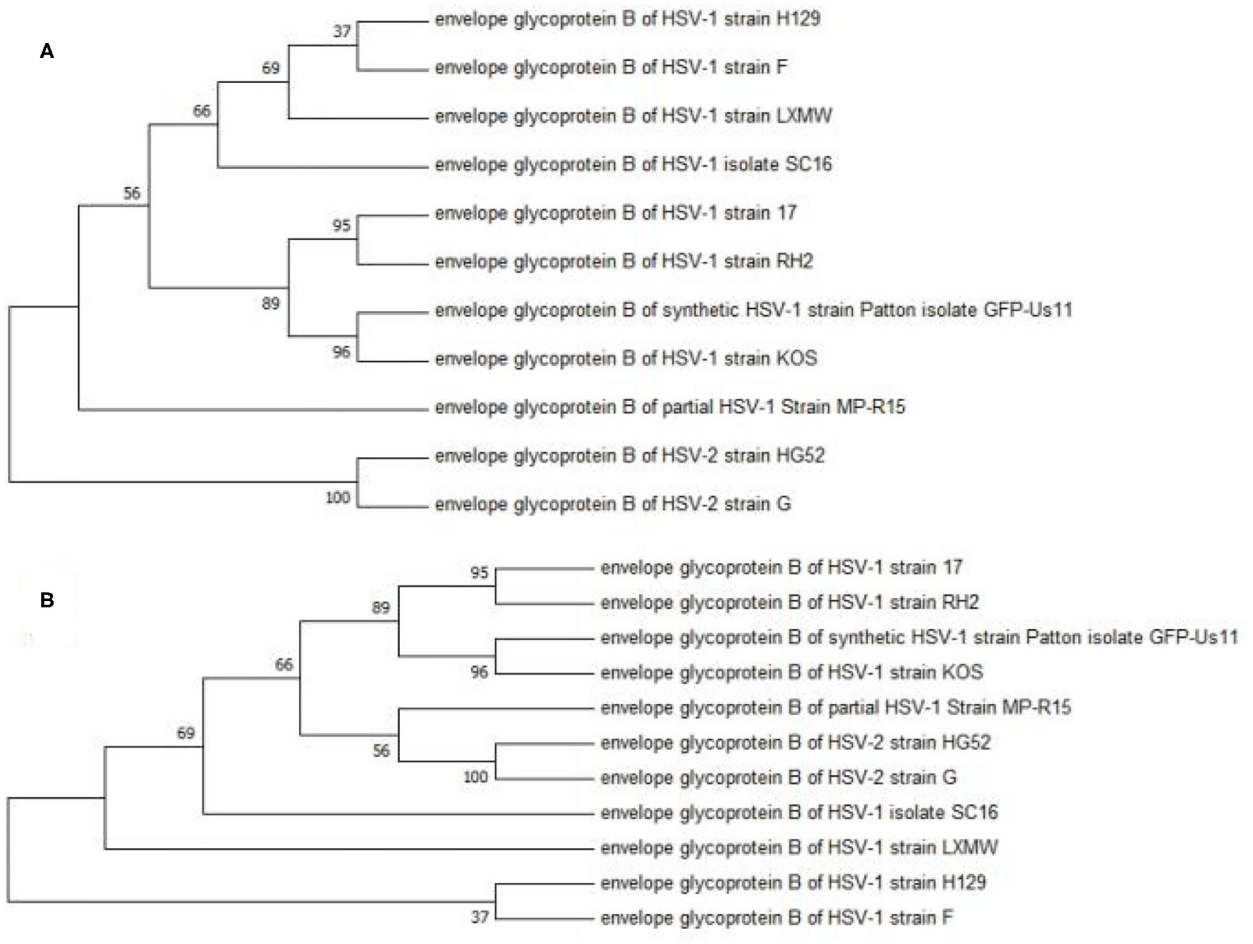
Phylogenetic analysis of the gBs of 11 HSV strains. This phylogenetic tree was generated by the neighbor-joining (NJ) method using MEGA7. The percentage of trees in which the associated taxa were clustered together is displayed next to the branches. The cladogram with the highest log likelihood is displayed. **(A)** The evolutionary tree is drawn to scale, with branch lengths measured in number of substitutions per site. **(B)** The bootstrap consensus tree is taken to represent the evolutionary history of the taxa analyzed. Our result showed an average distance of about 69% among the strains.

A phylogenetic analysis was performed to analyze the evolutionary relationship among the 11 HSV-1 strains. The *UL27* DNA sequence of HSV-1-LXMW, together with 11 HSV strains (Table 1), was translated into amino acid sequences and was then analyzed using the MEGA7 software. The results further supported that HSV-1-LXMW belongs to an HSV-1 strain, but not to an HSV-2 strain. Interestingly, the clades corresponding to HSV-1-LXMW and HSV-1 strains F and H129 were grouped together into one main branch in the phylogenetic trees, indicating a closer evolutionary link to each other likely due to their origin from a common ancestor (Figure 1). However, HSV-1-LXMW is grouped in a different branch from the HSV-1 strain KOS (Figure 1). Ultimately, the analysis suggests that the *UL27* gene of HSV-1-LXMW is evolutionarily close to HSV-1 strains F, H129, and SC16.

### Prediction of the Secondary Structure, Motifs, and Functional Domains of gB

#### The Prediction of gB Secondary Structure

**Figure 2:**
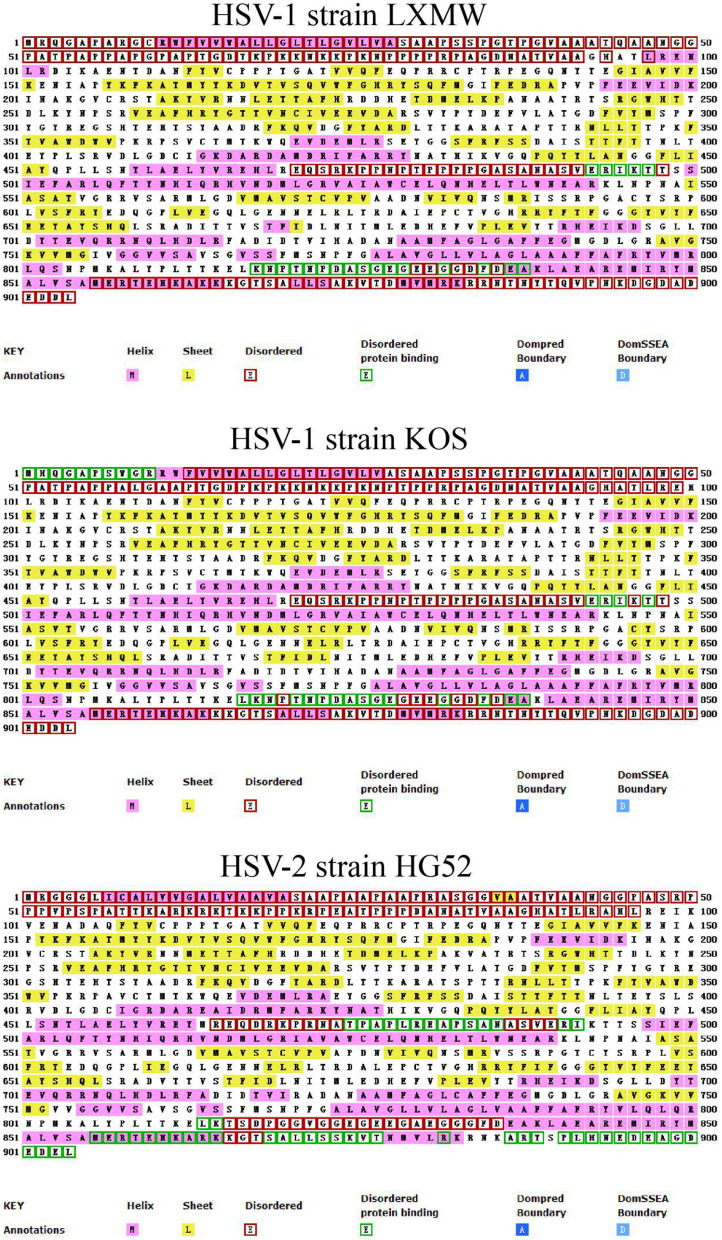
The predicted gB secondary structures of HSV-1 strains LXMW and KOS as well as HSV-2 strain HG52.

In order to analyze the secondary structure of gB and present it simply, we selected HSV-1 strains LXMW and KOS, and HSV-2 strain HG52, and predicted their secondary structure using the online software UCL-CS Bioinformatics (http://bioinf.cs.ucl.ac.uk/introduction/). As shown in Figure 2, the secondary structural forms of gB are various and abundant and include the helix, sheet, disordered, disordered protein binding, dompred boundary, and DomSSEA boundary formations. To be specific, the disordered protein binding sites in HSV-1 strain LXMW are in residues Glu493-Thr497 and residues Lys818-Ala838. For HSV-1 strain KOS, this site is located at residues Met1-Arg10, residues Glu493-Thr497, and residues Leu817-Ala838. However, for HSV-2 strain HG52, the site is located at residues Thr475-Ile492, Leu814-Lys815, Met856-Thr877, Arg882, and Ala887-Leu904.

#### The Prediction of 22 Novel gB Motifs From 11 HSV Strains

**Figure 3:**
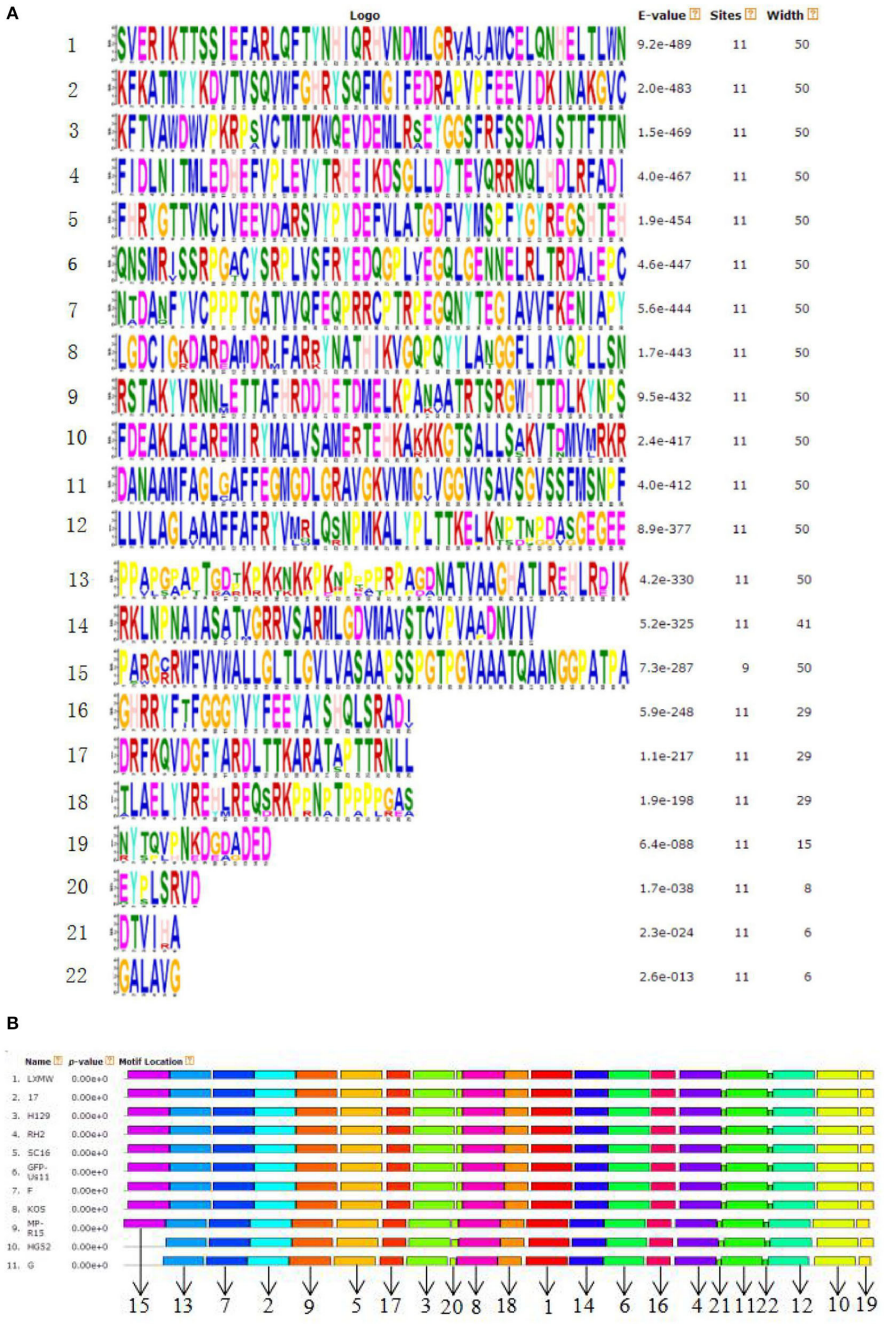
**(A)** The predicted consensus sequences of the 22 motifs from the gBs of 11 HSV strains. **(B)** The location of the 22 new gB motifs (1–21, 23) predicted in **(A)**.

To predict the gB motifs, the online software MEME was used (http://meme-suite.org/). The motif amino acid sequences were marked in Figure 3A and the newly predicted new motifs were named 1–22 in Figure 3B. The result revealed that HSV-2 gB lacked motif 15, located in Pro6 to Ala55. All HSV-1 and HSV-2 gBs share 21 motifs except motif 15 (Figures 3, **5**).

### gB Motifs Are Conserved Among 11 HSV Strains

**Figure 4:**
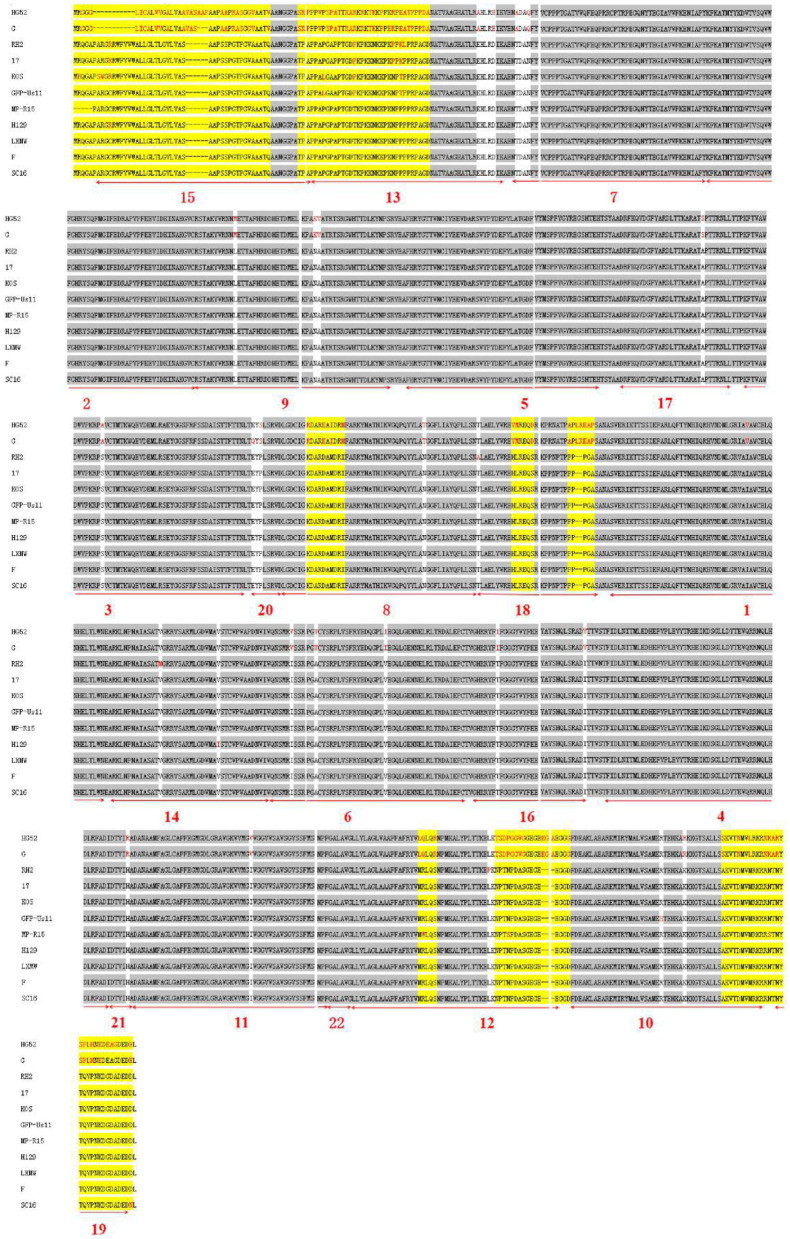
The gB amino acid sequence alignment of 11 HSV strains.

To further analyze the conservatism of gB, the gB amino acid sequences of the 11 HSVs were aligned (Figure 4). The alignment showed that there are 28 conserved and 8 variable regions in gB. Moreover, among the gBs of HSV-1 strains, there were fewer mutations among the gBs of HSV-1 strains. However, there were more variations between HSV-1 and HSV-2. In the gB domain of HSV-1 strain H129, two different amino acids (residues Arg10 and Ile570) were found from HSV-1 strain LXMW (residues Cys10, Val570, and Asp903). In the gB region of HSV-1 strain SC16, there was one different amino acid (residue Asn903) compared to HSV-1 strain LXMW. Both the phylogenic analysis and alignment corroborated the conclusion that gB amino acids of LXMW are highly similar to HSV-1 strains F, H129, and SC16.

For the 11 HSVs analyzed, the 28 conserved sequences among all the gB amino acids are marked by gray shade section, while the 8 variable amino acids are marked by yellow shade section. The different amino acids are marked in red. The 22 motifs predicted by the online software are marked by numerals 1–22 and underlined in bold red.

### The Potential Function of the 13 Newly Identified Motifs for gB of HSV

**Figure 5:**
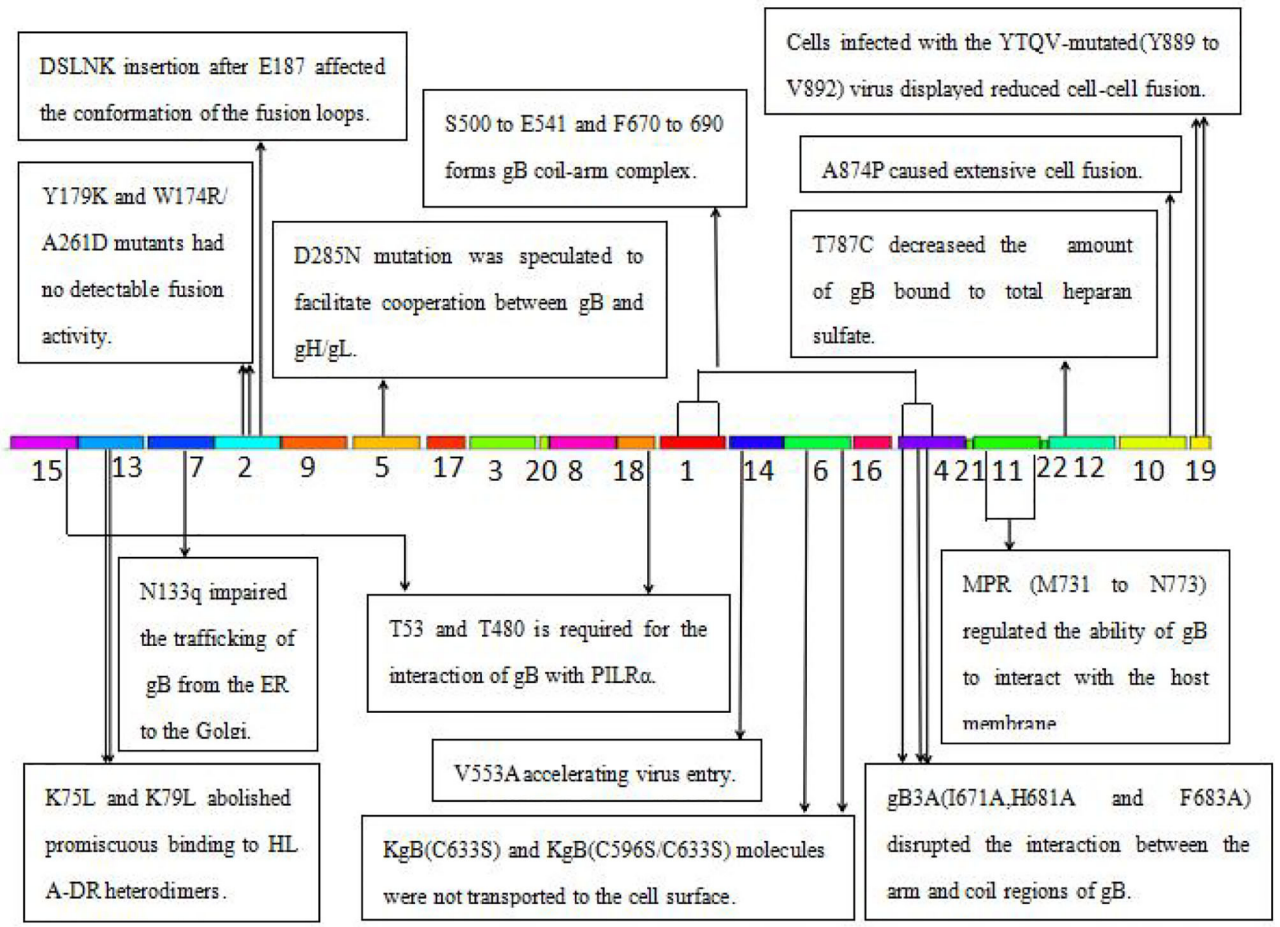
The function of the identified 22 motifs of gB of HSV. MPR, membrane-proximal region; PILR, the paired immunoglobulin-like type 2 receptor; HLA-DR, human leukocytic antigen DR; ER, endoplasmic reticulum.

It is well acknowledged that gB is a glycoprotein needed for HSV infection, which can form different dimers to mediate virus–cell fusion and induce the production of neutralizing antibodies (13). To understand the potential function of the identified gB motifs, we performed an extensive literature search on the topic of HSV gB. We summarized the gB mutants and gB-engineered oHSVs (Table 2 and Figure 5). Based on the following pieces of literatures, we, for the first time, systematically compared the relationship of the engineered sites in gB with the conserved regions, functional domains, and motifs, and suggested probable functions for 13 motifs of the total 22. However, we were not able to find any report on any of the other 9 motifs in published studies. Indeed, it is a double-edged sword to engineer HSV gBs. Some mutations and modifications will enhance its functionality while other manipulations will severely undermine it. A summary of these modifications is detailed below.

**Table 2:** The function of the identified motifs of gB of HSV.

**Virus**	**Name**	**Mutations in gB**	**Conserved/variable region in gB**	**Motif in gB**	**Motif potential function**	**oHSV effect**	**Additional modification**	**References**
HSV-1 strain KOS	Y179K, W174R A261D	Y179K, W174R A261D	Conserved region 4	2	Motif 2 may be necessary for virus–cell fusion.	Loss of fusion	None	(29)
HSV-1 (strain not mentioned)	T665i5, V667i5I671i5, L673i5 E187i5	MFKHT inserted after T665, MFKHV inserted after aa667, DLLNI inserted after aa671, NSLNI inserted after aa673, DSLNK inserted after aa187	Conserved region 22	2 and 4	Motif 2 is important for the conformation of the fusion loops.	Fusion deficient	None	(30)
HSV-1 strain KOS	I671A, H681A, F683A	I671A, H681A, F683A	Conserved region 22	4	Motif 4 may be required for virus–cell fusion possibly via interaction of the arm with coil regions of gB.	Decreased fusion	gH:lacked	(31)
HSV-1 strain F	T887A or truncated at residue 886.	T887A or truncated at residue 886.	Variable region 8	None		Reduced virus–cell fusion	gH:lacked	(32)
HSV-1 strain KOS	Y889A LL871AA	Disruption of YTQV motif (aa 889 to 892) and LL motif (aa 871 to 872)	Conserved region 28 and variable region 8	19 and 10	Motif 19 and 10 are involved in cell–cell fusion.	Disruption of YTQV and LL decrease and facilitate virus–cell fusion, respectively	None	(33)
HSV-1 strain KOS	gBΔ28, gBΔ36, A874P	gBΔ28, gBΔ36, A874P	Conserved region 28, variable region 8	10 and 19	Motifs 10 and 19 may participate in virus-induced fusion activity.	Increased cell fusion	None	(34)
HSV-1 strain F	syn3	R857H	Conserved region 26	10	Motif 10 may promote cell–cell fusion and this mutant effectively reduced primary and metastatic breast tumors in immunocompetent mice.	Effective in the treatment	gK: Ala-to-Val at position 40	(35)
HSV-1 strain KOS	Truncated at 863	Lacking the last 41 amino acids of the cytoplasmic domain	Conserved regions 27, 28, variable region 8	10 and 19	Deletion of the carboxy-terminal 41 amino acids had no influence on the function of gB.	No influence	None	(36)
HSV-1 strain F	R-903	Engineered the scFv to HER2 between AA 43 and 44	Variable region 1	15	Motif 15 may be either flexible or non-essential and oHSVs were capable to target Her2+ cancer cells	No influence	None	(22)
HSV-1	R-313, R-315, R-317, R-319	The GCN4 peptide inserted between aa 43 and 44, 81 and 82, 76 and 77, and 95 and 96	Conserved region 6	15 and 13	Motif 15 may be either flexible or non-essential and gB retargeted oHSV could cultivate in non-cancer cells.	No influence	gD: scFv-HER2 replaced aa 6 to 38	(37)
HSV-1 strain KOS	D285N/A549T	D285N/A549T	Conserved region 6 and 15	5 and 14	Motif 5 may regulate the interaction of gB with gH/gL, motif 14 may be essential for the conformational change of gB and the double mutant was effective in an orthotopic mouse model of primary human glioma.	Accelerate cells entry; facilitate cell fusion	gD: R222N/F223I gD: An scFv replaced aa 2–24	(38, 39)
HSV-1 strain KOS	S668N	S668N	Conserved region 21	None		gH/ gB formation	gH: Residues N753K and A778V in the H3 domain	(40)
HSV-1 strain F	T53A, T53/480A	T53A, T53/480A	Variable region 2, conserved region 13,	15 and 17	Motifs 15 and 17 may be required for the binding of gB to PILRα and virus–cell fusion.	Decreased virus–cell fusion and virus entry		(41)
HSV-1 strain S (a syncytial mutant from HSV-1strain F)	T787C	T787C of the cytoplasmic domain of mature gB, results in L to P substitution	Conserved region 24	12	Motif 12 may contribute to gB binding to heparan sulfate.	Decreased gB/heparan sulfate binding	None	(42)
HSV-1 strain KOS	gBpK^−^	Residues 68 to 76 (KPKKNKKPK) were deleted	Variable region 2	13	Motif 13 may be the heparan sulfate binding element of gB.		gC: gC coding sequence (UL44) deleted	(43)
HSV-1 strain 17	K76L K79L	Replace the aa 75PKPPKP80 with 75PLPPLP80	Variable region 8	13	Motif 13 may be involved in the interaction between gB and HLA-DR heterodimers.	Destroyed gB/HLA-DR heterodimers	None	(44)
HSV-1 strain KOS	C633S or C596S/C633S	Replace the aa 633C or both 633C and 596C with S	Conserved region 19, 24	6	Motif 6 may translocate gB to the cell surface.	Unable to translocate to the cell surface	None	(45)
HSV-2 strain G	N133Q	N133Q	Conserved region 4	7	Motif 7 may be required for gB trafficking from the ER to the Golgi.	Not transported from the ER to the Golgi	None	(46)
HSV-1 strain KOS	gBΔ(730–747), gBΔ(748–773), gBΔ(730–739), gBΔ(735–744), gBΔ(740–749), gBΔ(750–759), gBΔ(765–773)	gBΔ(730–747), gBΔ(748–773), gBΔ(730–739), gBΔ(735–744), gBΔ(740–749), gBΔ(750–759), gBΔ(765–773)	Conserved region 23,24	11	Motif 11 may be required for the interaction between gB and the cell membrane.	Cannot arrive at the cell surface	None	(47)

Several key motifs of gB play important roles in promoting virus–cell fusion. Sometimes, mutations in some motifs of gB could attenuate virus–cell fusion. Hannah BP et al. found that Y179K and W174R/A261D mutants hardly executed fusion activity (29). Residues Tyr179 and Trp174 are located in motif 2, suggesting that motif 2 is necessary for virus–cell fusion. Silverman JL et al. generated five insertion mutants (T665i5, V667i5, L673i5, I671i5, and E187i5) that caused a fusion-deficient phenotype. They speculated that the mutated region in E187i5 (in motif 2) was involved in the destruction of the fusion loops (30), suggesting that motif 2 is important for the conformation of the fusion loops. Connolly et al. found that three mutations (I671A, H681A, and F683A located in motif 4) in the arm region decreased fusion but did not reduce surface expression (31), suggesting that motif 4 is possibly required for virus–cell fusion via interaction of the arm with gB coil regions. Wisner TW et al. found that the T887A (not in any of the 22 motifs) mutation or truncation at Asn886 markedly reduced virus–cell fusion (32). Beitia et al. discovered that the destruction of YTQV (residues Tyr889 to Val892) decreased cell–cell fusion, but the damage of LL (residues Leu871 to Leu872) facilitated cell–cell fusion. They subsequently hypothesized that the LL mutations returned gB to the cell surface (33). This study suggested that motif 19 (Tyr889) and 10 (Leu871 to Leu872) are involved in cell–cell fusion.

However, there are also certain mutations in some motifs of gB-improved virus–cell fusion. Foster TP et al. concluded that the deletion of 28–36 amino acids in the carboxyl-terminal, or A874P mutation, increased virus–cell fusion, and the carboxyl-terminal 36 amino acids participated in membrane fusion (34). Since carboxyl-terminal 36 amino acids are distributed in motifs 10 and 19, these two motifs were also suggested to participate in virus–cell fusion. Israyelyan et al. demonstrated that fusogenic OncdSyn viruses, which relied on a combination of mutations R857H of gB (syn3) and gK (syn1), were effective in the treatment of breast tumors (35). Huff V et al. found that deletion of the carboxyl-terminal 41 amino acids had no influence on the function of gB (36). Petrovic B et al. inserted single-chain variable fragment (scFv) between residues 43 and 44 of gB, generating a mutant HSV R-903 that retargeted to HER2-positive cancer cells. They found that R-903 did not require activation by other glycoproteins (22). One year later, they inserted the GCN4 peptide into gB to construct a gB retargeted virus that could be cultivated in non-cancer cells (37). Residues 43 and 44 are located in motif 15, suggesting that motif 15 is either flexible or non-essential.

Some motifs are required for the attachment to gH/gL, PILRα, heparan sulfate, and HLA-DR. When gD receptors are nonexistent, gB double mutations D285N (in motif 5) and A549T (in motif 14) could allow HSV to enter into cells processed by facilitating interaction between gB and gH/gL and virus–cell fusion, respectively (38). In 2013, Uchida et al. established an oHSV that retargeted HSV to EGFR by combining the gD and gB mutations. This retargeting system was effective in the treatment of glioma (39). The D285N mutation is located in motif 5 and A549T mutation is located in motif 14. These results suggest that motif 5 may regulate gB interaction with gH/gL and motif 14 may be essential for gB conformational change (34). Uchida H et al. also showed that the lack of traditional gD receptors could also be offset by the combination of gH:KV and gB:S668N (not in any of the 22 motifs) (40). Arii et al. identified that gB-T53A (in motif 15) and gB-T53/480A (in motif 17) mutations distinctly decreased virus–cell fusion and virus entry into cells expressing PILRα (41), suggesting that motifs 15 and 17 are required for the binding of gB to PILRα as well as virus–cell fusion. Diakidi-Kosta et al. revealed that the T787C mutation decreased the amount of gB bound to total heparan sulfate (42). Residue Gly787 is located in motif 12, suggesting that motif 12 contributes to gB and heparan sulfate binding. Laquerre et al. concluded that the binding of gB to heparan sulfate required residues Lys68 to Lys76 (in motif 13) (43), implying that motif 13 is the heparan sulfate binding element of gB. Sievers et al. found that a combination of K76L and K79L (in motif 13) mutations destroyed the interaction between gB and HLA-DR heterodimers (44), suggesting that motif 13 is involved in the formation of gB and HLA-DR heterodimers.

Some motifs are involved in gB translocation and trafficking. Laquerre S et al. constructed KgB (C633S in motif 6) and KgB (C596S/C633S) mutant viruses that resulted in gB being unable to translocate to the cell surface. The mutant gB was not mature and therefore HSV could not infect cells (45). Residue Cys633 is located in motif 6, suggesting that motif 6 is required for gB translocating to the cell surface. Luo et al. demonstrated that the N133Q (Cys133 in motif 7) mutant was not transported from the ER to the Golgi, and therefore gB lost its function (46), suggesting that motif 7 is required for gB trafficking from the ER to the Golgi. Shelly et al. concluded that membrane-proximal region (Ala730 to Asn773 in motif 11) deletion mutants cannot arrive at the cell surface (47), suggesting that motif 11 is required for the gB trafficking to the cell membrane.

Taken together, key motifs can be modified in the aspects as follows to improve the efficiency of oHSV. First, the reasonable modification of motifs 2, 10, and 19 can increase virus–cell fusion. Second, to facilitate the translocation and trafficking of gB, modifications of motifs 6, 7, and 11 should be cautious. Third, motif 15 is flexible for exogenous gene fragment insertion, such as scFv-HER2, GCN4 peptide, etc. Furthermore, key motifs of gB determining antibody and complement neutralization could be explored and modified to evade immune response and improve efficacy of oHSV.

## Discussion

The development of more effective oHSVs depends on a deeper understanding of HSV gB structure and function. Here, we performed a systematic comparative sequence analysis of gBs from 9 HSV-1 and 2 HSV-2 strains, including our recently isolated new strain HSV-1-LXMW. Phylogenetic analysis found that HSV-1-LXMW gB is evolutionarily close to HSV-1 strains F, H129, and SC16. For the first time, we identified 22 novel motifs in HSV gB. Amino acid sequence alignment showed that the 22 motifs are conserved among HSV gBs. Through literature analysis, we found that 13 out of the 22 motifs are biologically functional. We also identified 28 conserved regions and 8 variable regions of HSV gB. Our findings may have significant implications on HSV gB biology and the construction of gB-engineered oHSVs with improved the efficacy and safety.

The phylogenetic analysis revealed that the gB amino acid sequence in HSV-1-LXMW was close to those in HSV-1 strains F, H129, and SC16 based on *UL27* gene analysis, while our earlier study demonstrated that ICP27 of our new strain is close to those in HSV-1 strains Patton and H129 (27). This study complements previous work demonstrating that our strain may be evolutionarily closer to strain H129. Therefore, any further manipulation done to LXMW strain's gB could benefit from the previously successful engineering strategies of gB from strain H129. The different genes may show some evidence of the phylogenetic relationships of different HSV-1 strains. When more genes are included in the analysis, more accurate phylogenetic relationships of different HSV-1 strains may be obtained.

We identified 22 motifs of HSV gB. Our investigations into previous literature revealed no reports on these motifs yet. Since the motifs were conserved among different HSVs, they are likely functional. Indeed, 13 of the 22 motifs are reported to be functional, which validated our newly identified motifs. However, the 13 motifs reported in the literature and the 9 unreported motifs must be further studied to concretely determine their functions, such as induction of neutralizing antibody responses. These fascinating novel functional motifs may be closely related to gB function and HSV infection, which could attract more and more attention in the future.

The alignment and comparison of different HSV gBs revealed conserved regions of gB that overlapped with some of the predicted motifs. This finding supports that the motifs are conserved and are more likely to be functional. Our analyses not only validated the function of the newly identified motifs in our new strain by using the previous reports but also provided the reference for future oHSV motif engineering in the future. Many of the motifs will be candidates as the sites of modification for generating new oHSVs. The previous functional studies may delete or mutate one or more amino acid(s) of gB randomly, while future functional studies on gB may delete the whole motif based on our work. For example, motif 19 (Tyr889) or motif 10 (Leu871–Leu872), instead of the point mutations, may be deleted to test motif function in virus–cell fusion in future studies (33).

Manipulating envelope glycoproteins of HSVs for oncolytic virotherapy would achieve strict targeting of tumor cells as well as complete de-targeting from healthy tissues to allow maximum oncolytic efficacy and minimum adverse effects. Ideally, such engineering should promote systemic administration of oHSVs, enabling direct targeting of not only the primary tumors but also distant metastatic cancer cells. Some studies showed that intravenous administration of oHSVs produced a significant reduction of primary tumors and was effective in treating metastatic tumors (48–52). Further preclinical and clinical trials are indispensable for the intravenous administration of oHSVs. In addition, although replication of oHSVs in cancer cells is a major determinant of therapeutic efficacy, activating the immune system to kill cancer cells is an important consideration. Targeting tumor specific antigens with modified gB, or with the other modified envelope glycoproteins, does not theoretically address the issue of tumor heterogeneity, which is considered a common cause of treatment failure for targeted therapy. Therefore, the development of multi-targets and combination therapy targeting different tumor cell subgroups is the topic of the research field in the future.

One of the most critical requirements for oHSV is tumor specificity. Although cancer specificity of many oHSVs has been described, the replication and killing specificity of oHSVs still have room to improve. Engineered gB can facilitate specific virus entry, trigger extensive cell fusion, enable oHSV cultivation in the non-cancer cells, and retarget oHSV to cancer cells in combination with gD (26, 31, 34, 43, 44). Although some strategies of manipulating envelope glycoproteins of HSV have been tested successfully in preclinical experiments (22, 37, 53), in oncolytic virotherapy, it remains to be determined whether the strategy of manipulating envelope glycoproteins of HSV is appropriate for clinical translation. Future studies will likely focus on the engineering of gB to ameliorate the efficacy and safety of oHSVs.

## Data Availability Statement

The original contributions presented in the study are included in the article/Supplementary Material, further inquiries can be directed to the corresponding authors.

## Author Contributions

FS: contributed to this paper with the design. FS, VX, X-QL, Y-YW, YZ, and J-TC: literature search. FS and W-QC: drafting. FS, XW, YX, and X-CP: revision. H-WX and XW: editing and final approval. All authors contributed to the article and approved the submitted version.

## Conflict of Interest

The authors declare that the research was conducted in the absence of any commercial or financial relationships that could be construed as a potential conflict of interest.
