# Supplementary material for: Identification of 22 Novel Motifs of the Cell Entry Fusion Glycoprotein B of Oncolytic Herpes Simplex Viruses: Sequence Analysis and Literature Review
Source: Front Oncol. 2020 Aug 19;10:1386. doi: 10.3389/fonc.2020.01386 (PMC7466406; doi:10.3389/fonc.2020.01386)
Supplement: Supplementary file 1 [file Data_Sheet_1.docx]

**Supplementary materials：**

The *UL27* DNA sequence:

**53088**TCACAGGTCGTCCTCGTCGGCGTCACCGTCTTTGTTGGGAACTTGGGTGTAGTTGGTGTTGCGGCGCTTGCGCATGACCATGTCGGTGACCTTGGCGCTAAGCAGCGCGCTCGTGCCCTTCTTCTTGGCCTTGTGTTCCGTGCGCTCCATGGCCGACACCAGGGCCATGTACCGTATCATCTCCCGGGCCTCGGCTAGCTTGGCCTCGTCAAAGTCGCCGCCCTCCTCGCCCTCCCCGGACGCGTCCGGGTTGGTGGGGTTCTTGAGCTCCTTGGTGGTTAGCGGGTACAGGGCCTTCATGGGGTTGCTCTGCAGCCGCATGACGTAGCGAAAGGCGAAGAAGGCCGCCGCCAGGCCGGCCAGGACCAACAGACCCACGGCCAGCGCCCCAAAGGGGTTGGACATGAAGGAGGACACGCCCGACACGGCCGATACCACGCCGCCCACGATGCCCATCACCACCTTGCCGACCGCGCGCCCCAGGTCGCCCATCCCCTCGAAAAACGCACCCAGGCCCGCGAACATGGCGGCGTTGGCGTCGGCGTGGATGACCGTGTCGATGTCGGCAAAGCGCAGGTCGTGCAGCTGGTTGCGGCGCTGGACCTCCGTGTAGTCCAGCAGGCCGCTGTCCTTGATCTCGTGGCGGGTGTACACCTCCAGGGGGACAAACTCGTGATCCTCCAGCATGGTGATGTTGAGGTCGATGAAGGTGCTGACGGTGGTGATGTCGGCGCGGCTCAGCTGGTGGGAGTACGCGTACTCCTCGAAGTACACGTAGCCCCCACCGAAGGTGAAGTAGCGCCGGTGTCCCACGGTGCACGGCTCGATCGCATCGCGCGTCAGCCGCAGCTCGTTGTTCTCCCCCAGCTGCCCCTCGACCAACGGGCCCTGGTCTTCGTACCGAAAGCTGACCAGGGGGCGGCTGTAGCAGGCCCCGGGCCGCGAGCTGATGCGCATCGAGTTTTGGACGATCACGTTGTCCGCGGCGACCGGCACGCACGTGGAGACGGCCATCACGTCGCCGAGCATCCGCGCGCTCACCCGCCGGCCCACGGTGGCCGATGCGATGGCGTTGGGGTTCAGCTTGCGGGCCTCGTTCCACAGGGTCAGCTCGTGATTCTGCAGCTCGCACCACGCGATGGCAACGCGGCCCAACATATCGTTGACATGGCGCTGTATGTGGTTGTACGTAAACTGCAGCCGGGCGAACTCGATGGAGGAGGTGGTCTTGATGCGCTCCACGGACGCGTTGGCGCTGGCCCCGGGCGGCGGGGGCGTGGGGTTTGGGGGCTTGCGGCTCTGCTCTCGGAGGTGTTCCCGCACGTACAGCTCCGCGAGCGTGTTGCTGAGAAGGGGCTGGTACGCGATCAGAAAGCCCCCATTGGCCAGGTAGTACTGCGGCTGGCCCACCTTGATGTGCGTCGCGTTGTACCTGCGGGCGAAGATGCGGTCCATGGCGTCGCGGGCGTCCTTGCCGATGCAGTCCCCCAGGTCCACGCGCGAGAGCGGGTACTCGGTCAGGTTGGTGGTGAAGGTGGTGGATATGGCGTCGGAGGAGAATCGGAAGGAGCCGCCGTACTCGGAGCGCAGCATCTCGTCCACCTCCTGCCACTTGGTCATGGTGCAGACCGACGGGCGCTTTGGCACCCAGTCCCAGGCCACGGTGAACTTGGGGGTCGTGAGCAGGTTCCGGGTGGTCGGCGCCGTGGCCCGGGCCTTGGTGGTGAGGTCGCGCGCGTAGAAGCCGTCGACCTGCTTGAAGCGGTCGGCGGCGTAGCTGGTGTGTTCGGTGTGCGACCCCTCCCGGTAGCCGTAAAACGGGGACATGTACACAAAGTCGCCAGTCGCCAGCACAAACTCGTCGTACGGGTACACCGAGCGCGCGTCCACCTCCTCGACGATGCAGTTTACCGTCGTCCCGTACCGGTGGAACGCCTCCACCCGCGAGGGGTTGTACTTCAGGTCGGTGGTGTGCCAGCCCCGGCTCGTGCGGGTCGCGGCGTTGGCCGGTTTCAGCTCCATGTCGGTCTCGTGGTCGTCCCGGTGAAACGCGGTGGTCTCCAGGTTGTTGCGCACGTACTTGGCCGTGGACCGACAGACCCCCTTGGCGTTGATCTTGTCGATCACCTCCTCGAAGGGGACGGGGGCGCGGTCCTCAAAGATCCCCATAAACTGGGAGTAGCGGTGGCCGAACCACACCTGCGAAACGGTGACGTCTTTGTAGTACATGGTGGCCTTGAACTTGTACGGGGCGATGTTCTCCTTGAAGACCACCGCGATGCCCTCCGTGTAGTTCTGACCCTCGGGCCGGGTCGGGCAGCGGCGCGGCTGCTCGAACTGCACCACCGTGGCGCCCGTGGGGGGTGGGCACACGTAAAAGTTTGCATCGGTGTTCTCCGCCTTGATGTCCCGCAGGTGCTCGCGCAGGGTGGCGTGGCCCGCGGCGACGGTCGCGTTGTCGCCGGCGGGGCGCGGCGGCGGTGGGTTTTTCGGTTTTTTGTTCTTCTTCGGTTTCGTGTCCCCCGTTGGGGCGGGGCCAGGGGCGGGCGGCGCCGGAGTGGCAGGTCCCCCGTTCGCCGCCTGGGTCGCGGCCGCGACCCCAGGCGTGCCGGGGGAACTCGGAGCCGCCGACGCCACCAGGACCCCCAGCGTCAACCCCAAGAGCGCCCATACGACGAACCACCGGCACCCCCGCGCGGGGGCGCCCTGGCGCAT**55802**

*The gB amino acids sequence:*

MRQGAPARGCRWFVVWALLGLTLGVLVASAAPSSPGTPGVAAATQAANGGPATPAPPAPGPAPTGDTKPKKNKKPKNPPPPRPAGDNATVAAGHATLREHLRDIKAENTDANFYVCPPPTGATVVQFEQPRRCPTRPEGQNYTEGIAVVFKENIAPYKFKATMYYKDVTVSQVWFGHRYSQFMGIFEDRAPVPFEEVIDKINAKGVCRSTAKYVRNNLETTAFHRDDHETDMELKPANAATRTSRGWHTTDLKYNPSRVEAFHRYGTTVNCIVEEVDARSVYPYDEFVLATGDFVYMSPFYGYREGSHTEHTSYAADRFKQVDGFYARDLTTKARATAPTTRNLLTTPKFTVAWDWVPKRPSVCTMTKWQEVDEMLRSEYGGSFRFSSDAISTTFTTNLTEYPLSRVDLGDCIGKDARDAMDRIFARRYNATHIKVGQPQYYLANGGFLIAYQPLLSNTLAELYVREHLREQSRKPPNPTPPPPGASANASVERIKTTSSIEFARLQFTYNHIQRHVNDMLGRVAIAWCELQNHELTLWNEARKLNPNAIASATVGRRVSARMLGDVMAVSTCVPVAADNVIVQNSMRISSRPGACYSRPLVSFRYEDQGPLVEGQLGENNELRLTRDAIEPCTVGHRRYFTFGGGYVYFEEYAYSHQLSRADITTVSTFIDLNITMLEDHEFVPLEVYTRHEIKDSGLLDYTEVQRRNQLHDLRFADIDTVIHADANAAMFAGLGAFFEGMGDLGRAVGKVVMGIVGGVVSAVSGVSSFMSNPFGALAVGLLVLAGLAAAFFAFRYVMRLQSNPMKALYPLTTKELKNPTNPDASGEGEEGGDFDEAKLAEAREMIRYMALVSAMERTEHKAKKKGTSALLSAKVTDMVMRKRRNTNYTQVPNKDGDADEDDL
